# Iodate Reduction by *Shewanella oneidensis* Requires Genes Encoding an Extracellular Dimethylsulfoxide Reductase

**DOI:** 10.3389/fmicb.2022.852942

**Published:** 2022-04-14

**Authors:** Hyun-Dong Shin, Yael Toporek, Jung Kee Mok, Ruri Maekawa, Brady D. Lee, M. Hope Howard, Thomas J. DiChristina

**Affiliations:** ^1^Georgia Institute of Technology, School of Biological Sciences, Atlanta, GA, United States; ^2^School of Materials, Chemistry and Chemical Engineering, Osaka Prefecture University, Sakai, Japan; ^3^Savannah River National Laboratory, Environmental Sciences Section, Aiken, SC, United States

**Keywords:** iodate reduction, radioactive iodine, bioremediation, DMSO reductase, *Shewanella oneidensis*, anaerobic respiration, formate metabolism, molybdopterin

## Abstract

Microbial iodate (IO_3_^–^) reduction is a major component of the iodine biogeochemical reaction network in anaerobic marine basins and radioactive iodine-contaminated subsurface environments. Alternative iodine remediation technologies include microbial reduction of IO_3_^–^ to iodide (I^–^) and microbial methylation of I^–^ to volatile gases. The metal reduction pathway is required for anaerobic IO_3_^–^ respiration by the gammaproteobacterium *Shewanella oneidensis*. However, the terminal IO_3_^–^ reductase and additional enzymes involved in the *S. oneidensis* IO_3_^–^ electron transport chain have not yet been identified. In this study, gene deletion mutants deficient in four extracellular electron conduits (EECs; Δ*mtrA*, Δ*mtrA*-Δ*mtrDEF*, Δ*mtrA*-Δ*dmsEF*, Δ*mtr*A-ΔSO4360) and DMSO reductase (Δ*dmsB*) of *S. oneidensis* were constructed and examined for anaerobic IO_3_^–^ reduction activity with either 20 mM lactate or formate as an electron donor. IO_3_^–^ reduction rate experiments were conducted under anaerobic conditions in defined minimal medium amended with 250 μM IO_3_^–^ as anaerobic electron acceptor. Only the Δ*mtrA* mutant displayed a severe deficiency in IO_3_^–^ reduction activity with lactate as the electron donor, which suggested that the EEC-associated decaheme cytochrome was required for lactate-dependent IO_3_^–^ reduction. The Δ*mtrA*-Δ*dmsEF* triple mutant displayed a severe deficiency in IO_3_^–^ reduction activity with formate as the electron donor, whereas Δ*mtrA*-Δ*mtrDEF* and Δ*mtr*A-ΔSO4360 retained moderate IO_3_^–^ reduction activity, which suggested that the EEC-associated dimethylsulfoxide (DMSO) reductase membrane-spanning protein DmsE, but not MtrA, was required for formate-dependent IO_3_^–^ reduction. Furthermore, gene deletion mutant Δ*dmsB* (deficient in the extracellular terminal DMSO reductase protein DmsB) and wild-type cells grown with tungsten replacing molybdenum (a required co-factor for DmsA catalytic activity) in defined growth medium were unable to reduce IO_3_^–^ with either lactate or formate as the electron donor, which indicated that the DmsAB complex functions as an extracellular IO_3_^–^ terminal reductase for both electron donors. Results of this study provide complementary genetic and phenotypic evidence that the extracellular DMSO reductase complex DmsAB of *S. oneidensis* displays broad substrate specificity and reduces IO_3_^–^ as an alternate terminal electron acceptor.

## Introduction

Iodine is commonly found in the environment in the forms of iodide (I^–^; -1 oxidation state), iodate (IO_3_^–^;+5 oxidation state), and organic iodine compounds (Whitehead, 1984; Amachi, 2013; Kaplan et al., 2014; Fuge and Johnson, 2015). An unstable radioisotope of iodine, ^129^I, is a nuclear waste product produced during uranium and plutonium fission reactions and displays a long half-life of 16 million years (Timar et al., 2014). ^129^I is found in contaminated groundwater at the U.S. Department of Energy Savannah River and Hanford Superfund sites from a long history of nuclear weapons testing (Emerson et al., 2014; Timar et al., 2014; Bagwell et al., 2019). Following the 2011 Fukushima nuclear reactor catastrophe, westerly winds deposited a large portion of ^129^I in the Pacific Ocean, where radioactive IO_3_^–^ and I^–^ are the predominant ^129^I forms (Hou et al., 2007, 2013; Bluhm et al., 2011; Kenyon et al., 2020). IO_3_^–^ is more thermodynamically stable in seawater than I^–^; however, significant quantities of I^–^ are detected in anaerobic environments such as anaerobic basins and oxygen minimum zones in marine environments, which potentially indicates that microbial IO_3_^–^ reduction is a major component of the iodine biogeochemical reaction network (Whitehead, 1984; Councell et al., 1997; Farrenkopf et al., 1997; Wong et al., 2002; Chance et al., 2007; Bluhm et al., 2010; Amachi, 2013; Kaplan et al., 2014; Fuge and Johnson, 2015; Guido-Garcia et al., 2015). Microbial IO_3_^–^ reduction has also received recent attention as a component of alternative strategies for the remediation of waters and sediments contaminated with radioactive iodine inadvertently released to the environment (Amachi, 2013; Kaplan et al., 2014; Riley et al., 2016; Mok et al., 2018; Toporek et al., 2019). The presence of environmental ^129^I presents a significant risk of bioaccumulation in the human thyroid gland, as iodine is a biologically active element for humans and vertebrate animals as a constituent of the thyroid hormones, thyroxine, and triiodothyronine (Whitehead, 1984; Fuge and Johnson, 2015). Despite the human health concerns that surround the fate and transport of radioactive iodine in the environment, the molecular mechanism of microbial IO_3_^–^ reduction remains poorly understood in the iodine biogeochemical cycle (Amachi, 2013; Gong and Zhang, 2013; Kaplan et al., 2014; Fuge and Johnson, 2015; Yeager et al., 2017).

Several IO_3_^–^-reducing microorganisms have been reported, which include *Shewanella putrefaciens*, *Shewanella oneidensis*, *Desulfovibrio desulfuricans*, *Pseudomonas* sp. strain SCT, and *Rhizobiaceae* bacterium strain DVZ35 (Councell et al., 1997; Farrenkopf et al., 1997; Amachi et al., 2007; Mok et al., 2018; Toporek et al., 2019). In particular, the facultative anaerobe *S. oneidensis* reduces a wide range of terminal electron acceptors, which includes oxidized forms of iron, manganese, nitrogen, sulfur, uranium, plutonium, technetium, and iodine (Farrenkopf et al., 1997; Venkateswaran et al., 1999; Neu et al., 2005; Newsome et al., 2014; Mok et al., 2018; Toporek et al., 2019). *S. oneidensis* transfers electrons to a variety of extracellular electron acceptors, which include Mn(III) and Fe(III) and Mn(IV) oxides (Cooper et al., 2016; White et al., 2016). To transfer the electrons to external metal oxides, *S. oneidensis* employs a variety of novel respiratory strategies, which include (i) direct enzymatic reduction *via* decaheme *c*-type cytochromes associated with extracellular electron conduits (EECs) located on the surface or surface extensions of the *S. oneidensis* outer membrane (Myers and Myers, 1992; DiChristina et al., 2002; Gorby et al., 2006), (ii) extracellular electron transfer *via* endogenous or exogenous electron shuttling compounds (Taillefert et al., 2007; Fennessey et al., 2010; Jones et al., 2010), and (iii) non-reductive Fe(III) solubilization by organic ligands to produce more readily reducible soluble organic Fe(III) complexes (Hernandez et al., 2004; Marsili et al., 2008; Roden et al., 2010).

The previous studies of other IO_3_^–^-reducing microorganisms indicated that nitrate (NO_3_^–^) reductase may catalyze the reduction of IO_3_^–^ as an alternative electron acceptor (Tsunogai and Sase, 1969; Wong and Hung, 2001; Lee et al., 2018). However, results from the later studies indicated that neither assimilatory nor dissimilatory NO_3_^–^ reductases are required for IO_3_^–^ reduction by *S. oneidensis* (Mok et al., 2018). Recently, a putative periplasmic molybdopterin-dependent iodate reductase (Idr) system composed of four proteins (IdrA, IdrB, IdrP1, and IdrP2) was identified in *Pseudomonas* sp. strain SCT. The catalytic subunits IdrA and IdrB displayed amino acid sequence homology with the catalytic and electron transfer subunits of respiratory arsenite oxidase (Aio), and IdrA represented a novel clade within the dimethylsulfoxide (DMSO) reductase family (Yamazaki et al., 2020). Another estuarine bacterium, *Denitromonas* sp. IR-12, was also recently reported to utilize a molybdenum (Mo)-dependent IrdA for dissimilatory IO_3_^–^ reduction (Reyes-Umana et al., 2022).

**FIGURE 1:**
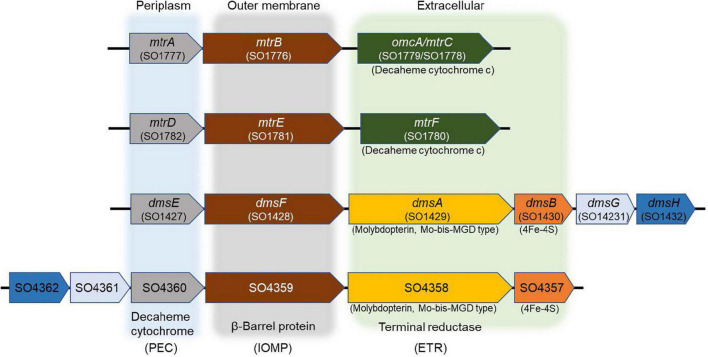
Mtr paralogs of extracellular electron conduits (EEC) system responsible for anaerobic respiration in *S. oneidensis.* PEC, periplasmic electron carrier; IOMP, integral outer membrane β-barrel protein; ETR, extracellular terminal reductase.

The IO_3_^–^ reduction pathway of *S. oneidensis* shares electron transport components with EEC systems that reduce alternate electron acceptors such as metals, NO_3_^–^, sulfur compounds, DMSO, and trimethylamine *N*-oxide (Myers and Myers, 1992; DiChristina et al., 2002; Hernandez et al., 2004; Gorby et al., 2006; Taillefert et al., 2007; Marsili et al., 2008; Fennessey et al., 2010; Jones et al., 2010; Roden et al., 2010; Cooper et al., 2016; White et al., 2016). The electron transport pathways of *S. oneidensis* consist of upstream dehydrogenases linked *via* the menaquinone pool and the inner membrane-tethered *c*-type cytochrome CymA to downstream terminal reductase complexes associated with the metal-reducing EEC (Marsili et al., 2008; Roden et al., 2010; Gong and Zhang, 2013). The metal-reducing EEC of *S. oneidensis* is comprised of outer membrane β-barrel protein MtrB (and essential cysteine residue C42) (Wee et al., 2014) and decaheme *c*-type cytochromes, MtrA and MtrC (Shi et al., 2006, 2012; Coursolle and Gralnick, 2010; Richardson et al., 2012; Richter et al., 2012; Szeinbaum et al., 2014). MtrC is translocated to the outside face of the outer membrane through GspD, the outer membrane secretin of the type II protein secretion system (Cianciotto, 2005; McLaughlin et al., 2012; Cooper et al., 2016). Other proteins that are essential for electron transport to external metal oxides include the *c*-type cytochrome maturation permease CcmB (Dale et al., 2007) and the cAMP receptor protein CRP required for anaerobic respiratory gene expression by *S. oneidensis* (Saffarini et al., 2003). On the other hand, *S. oneidensis* EEC-associated DMSO reductase DmsAB is comprised of DmsA, which requires the Mo-binding co-factor molybdopterin, and the ferredoxin subunit DmsB, which contains [4Fe-4S] clusters as co-factors (Figure 1; May et al., 1988; Gralnick et al., 2006).

The previous work suggests that outer membrane (type II) protein secretion and metal reduction genes encoding the outer membrane MtrAB module of the EEC MtrCAB are required for IO_3_^–^ reduction by *S. oneidensis* with lactate, but not formate, as the electron donor (Toporek et al., 2019). However, the metal-reducing *c*-type cytochrome MtrC associated with the EEC MtrAB was not required for IO_3_^–^ reduction by *S. oneidensis* with any electron donor tested. These findings indicate that the IO_3_^–^ electron transport pathway is modular, electron donor-dependent, and terminates with an as yet unidentified IO_3_^–^ reductase that associates with an outer membrane EEC to deliver electrons extracellularly to IO_3_^–^ (Toporek et al., 2019).

In addition to MtrCAB, the *S. oneidensis* genome harbors three additional gene clusters that encode the EECs MtrDEF, DmsEFAB, and SO4357-4360 (Bucking et al., 2010; Coursolle and Gralnick, 2010). MtrCAB and DmsEFAB are required for anaerobic reduction of Fe(III) and DMSO, respectively (Bucking et al., 2010; Coursolle and Gralnick, 2010; Schicklberger et al., 2013). Furthermore, several Mtr and Dms paralogs are functionally interchangeable (Coursolle and Gralnick, 2010, 2012; Schicklberger et al., 2013). For example, MtrD and DmsE may functionally replace MtrA (Coursolle and Gralnick, 2010), while MtrF and to a partial extent OmcA may functionally replace MtrC (Coursolle and Gralnick, 2010). MtrDEF reduces Fe(III) citrate at approximately half the rate of MtrCAB in ΔMtr mutants (Coursolle and Gralnick, 2012). While the expression of SO4359 and SO4360 alone was sufficient to complement an *mtrB* mutant under Fe(III) citrate-reducing conditions (Schicklberger et al., 2013). These findings led us to hypothesize that *S. oneidensis* reduces IO_3_^–^ with separate lactate (MtrAB)- and formate-dependent EEC paralogs that deliver electrons extracellularly to IO_3_^–^ (i.e., function as electron donor-dependent IO_3_^–^ terminal reductases).

The main objective of this study was to identify the IO_3_^–^ reductase of *S. oneidensis*. The experimental strategy consisted of the following steps: (i) construction of three EEC paralog mutants *via* the deletion of *mtrDEF*, *dmsEF*, and SO4360 in a Δ*mtrA* mutant host strain, and subsequent testing for IO_3_^–^ reduction activity; (ii) replacement of Mo with tungsten (W) in defined growth medium and tests for IO_3_^–^ reduction activity; and (iii) construction of DMSO reductase mutant Δ*dmsB* (deficient in DMSO reductase protein DmsB) and tests of Δ*dmsB* for IO_3_^–^ reduction activity with formate and lactate as the electron donor.

## Materials and Methods

### Growth and Cultivation Conditions

*Shewanella oneidensis* strains were routinely cultured aerobically at 30°C in lysogeny broth (LB) (10 g/L^–1^ of NaCl, 10 g/L^–1^ of tryptone, and 5 g/L^–1^ of yeast extract). IO_3_^–^ reduction rate experiments were conducted under anaerobic conditions in M1 minimal medium (Myers and Nealson, 1988; Supplementary Material) amended with 20 mM sodium formate or lactate as the electron donor and 250 μM IO_3_^–^ as the anaerobic electron acceptor. Anaerobic growth of MR-1 on iodate is minimal under the incubation conditions used in this study. The toxicity threshold of MR-1 to iodate concentrations was previously determined in defined medium under aerobic and anaerobic incubation conditions (Toporek et al., 2019). When required for selection, gentamicin (20 μg ml^–1^) or chloramphenicol (25 μg ml^–1^) was amended to the appropriate growth medium for the selection of deletion mutant or the maintenance of recombinant plasmid vector pBBR1MCS (Kovach et al., 1995).

### In-Frame Gene Deletion Mutagenesis

The genes *mtrDEF*, *dmsEF*, *SO4360*, and *dmsB* were deleted in frame from the *S. oneidensis* genome by following the previously described procedures (Supplementary Table 1; Burns and DiChristina, 2009). A *dmsA* deletion mutant was attempted but it was unsuccessful. Regions corresponding to 750 bp upstream and downstream of *mtrDEF*, *dmsEF*, *SO4360*, and *dmsB* were PCR amplified with Q5 High-Fidelity DNA polymerase (New England BioLabs, Ipswich, MA, United States) [primers D1/D2 and D3/D4 (Supplementary Table 2)] and subsequently joined using overlap extension PCR [primers D1/D4 (Supplementary Table 2)]. To construct Δ*mtrA*-Δ*mtrDEF*, Δ*mtrA*-Δ*dmsEF*, and Δ*mtrA*-Δ*SO4360* mutants, the resulting fragments of *mtrDEF*, *dmsEF*, and *SO4360* were cloned into suicide vector pKO2.0 (which does not replicate in *S. oneidensis*) and mobilized into *S. oneidensis* Δ*mtrA* (Szeinbaum et al., 2014) *via* conjugation with *E. coli* donor strain β2155 λ *pir* (Chung et al., 1989). In addition, the resulting fragment of *dmsB* was also cloned into suicide vector pKO2.0 and mobilized into wild-type *S. oneidensis via* conjugation with *E. coli* donor strain β2155 λ *pir* (Chung et al., 1989) to construct mutant Δ*dmsB*. *S. oneidensis* strains with the integrated plasmid were selected on LB agar containing gentamicin (20 μg ml^–1^). Single crossover integrations were verified using PCR with primers flanking the recombination region (TF/TR) and were resolved from the genomes by plating on LB agar lacking NaCl and containing sucrose [10% (wt/vol)]. The in-frame deletion strains (Δ*mtrA*-Δ*mtrDEF*, Δ*mtrA*-Δ*dmsEF*, Δ*mtrA*-Δ*SO4360*, and Δ*dmsB*) were verified by PCR with primers TF/TR (Supplementary Table 2). Genetic complementation analysis of the Δ*dmsB* strain was carried out by cloning the wild-type gene (after amplification from the *S. oneidensis* genome using primer set dB-F and dB-R; Supplementary Table 2) into broad-host-range cloning vector pBBR1MCS (Kovach et al., 1995) and conjugally transferring the recombinant vector into the respective mutant strains *via* biparental mating procedures (Chung et al., 1989).

### IO_3_^–^ and Dimethylsulfoxide Reduction Activity Assays

Mutant strains were initially inoculated in the liquid LB growth medium and incubated at 30°C for 24 h. About 10 ml of subcultures at an initial optical density at 600 nm (OD_600_) of 0.02 was incubated at 30°C for 24 h. Subcultures were centrifuged at 4,000 rpm for 30 min, resuspended in 10 ml of M1 growth medium amended with 10 mM formate and incubated aerobically at room temperature for 8 h. The preconditioned cells were inoculated in the 30-ml serum bottles at an initial OD_600_ of 0.1 in M1 growth medium amended with either 40 mM DMSO or 250 μM IO_3_^–^ and 10 mM formate and incubated anaerobically *via* continuous sparging with 100% high-purity (hydrated) N_2_ gas. Cultures were incubated at room temperature with gentle stirring under anaerobic conditions maintained by continuous sparging with high-purity hydrated N_2_ gas. At preselected time points, OD_600_ was measured and IO_3_^–^ concentrations were determined using the IO_3_^–^-triiodide formation method (Afkhami et al., 2001; Mok et al., 2018; Toporek et al., 2019) described below. Cells corresponding to OD = 0.1 contain 50 mg protein as measured by the Bradford assay (Bradford, 1976). DMSO reduction was monitored by measuring anaerobic growth at OD_600_. For substitution of molybdenum Mo with tungsten W in anaerobic IO_3_^–^ reduction activity assays, Mo was replaced with equal molar concentration of W in M1 medium, and IO_3_^–^ reduction activity was compared to IO_3_^–^-reduction activity in normal M1 medium containing Mo. For the cultivation of recombinant strains carrying pBBR1MCS or pBBR1MCS-*dmsB*, 25 μg ml^–1^ chloramphenicol and 0.1 mM IPTG were amended to the medium to maintain the plasmid and induce cloned *dmsB* gene expression, respectively.

### Determination of IO_3_^–^ Concentrations *via* IO_3_^–^-Triiodide Formation With I^–^ at Acidic pH

The extent of IO_3_^–^ reduction was determined using the IO_3_^–^-triiodide method (Afkhami et al., 2001; Mok et al., 2018; Toporek et al., 2019). Culture samples were added to the 96-well 500-μl microtiter plates. Sodium citrate buffer (0.1 M; pH 3.3) and potassium iodide solution (75 mM) were added to each well to initiate triiodide formation (IO_3_^–^ + 5I^–^ + 6H^+^ Δ 3H_2_O + 3I_2_). Absorbance at 352 nm was measured with a UV spectrophotometer (Multiskan Go; Thermo Scientific) within the first 3 min of reaction time. IO_3_^–^ concentrations were determined from a previously generated calibration curve.

## Results

### IO_3_^–^ Reduction Activity of Extracellular Electron Conduit Mutant Strains

To test the hypothesis that *S. oneidensi*s employs periplasmic and outer membrane proteins other than MtrAB to deliver electrons to IO_3_^–^, three additional EEC gene mutant strains (Δ*mtrDEF*, Δ*dmsEF*, and ΔSO4360) were constructed in a Δ*mtrA* mutant host strain. A Δ*mtrA* gene deletion background was selected since Δ*mtrA* retained wild-type IO_3_^–^ reduction activity with formate as electron donor (Toporek et al., 2019). Mtr proteins and their paralogs (e.g., DMS operon) are modular and can provide partial compensation for each other in the absence of a primary component (Coursolle and Gralnick, 2010). To avoid the possibility of MtrA compensating for the lack of DmsE, we constructed all *dms* mutants with a Δ*mtr*A background. The IO_3_^–^ reduction activities of the three additional EEC mutant strains (Δ*mtrA*Δ*mtrDEF*,Δ*mtrA*Δ*dmsEF*, and Δ*mtrA*Δ*SO4360*) were determined with either lactate or formate as the electron donor.

**FIGURE 2:**
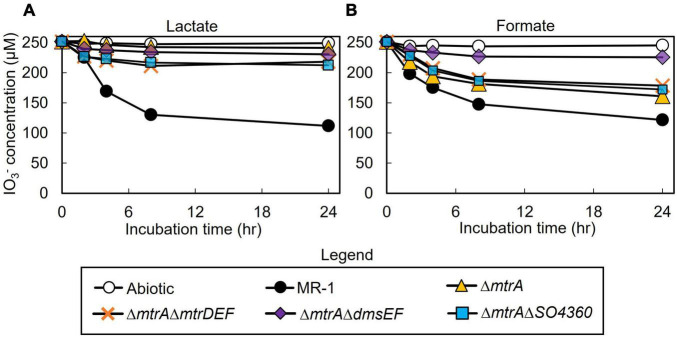
IO_3_^–^ reduction activity of *Shewanella oneidensis* wild-type (MR-1) and Δ*mtrA*, Δ*mtrA*Δ*mtrDEF*,Δ*mtrA*Δ*dmsEF*, and Δ*mtrA*Δ*SO4360* mutants with IO_3_^–^ as the electron acceptor and lactate **(A)** or formate **(B)** as the electron donor and their IO_3_^–^ reduction rate. Values are means of triplicate samples from anaerobic incubations. Error bars represent one standard deviation.

All mutant strains cultured with lactate displayed severely impaired (between 0 and 50% of wild-type activity) IO_3_^–^ reduction activities when compared to wild-type rates (Δ*mtrA*, 7 and 7% of the wild-type rate and extent of reaction, respectively; Δ*mtrA*Δ*mtrDEF*, 38 and 24% of the wild-type rate and extent of reaction, respectively; Δ*mtrA*Δ*dmsEF*, 17 and 15% of the wild-type rate and extent of reaction, respectively; and Δ*mtrA*Δ*SO4360*, 35 and 28% of the wild-type rate and extent of reaction, respectively; Table 1 and Figure 2). These results further confirm that EEC component MtrA is required for IO_3_^–^ reduction with lactate as the electron donor (Toporek et al., 2019; Figure 1).

**TABLE 1:** IO_3_^–^ reduction activities of wild-type and EEC paralog mutant strains of *Shewanella oneidensis* with lactate and formate as electron donors.^a^

Condition or strain	Electron donor: lactate		Electron donor: formate	
		
	IO_3_^–^ reduction rate^b^ (nmol hr^–1^ mg protein^–1^)^d^	Extent of reaction^c^ (% of IO_3_^–^ reduced to I^–^)^d^	IO_3_^–^ reduction rate^b^ (nmol hr^–1^ mg protein^–1^)^d^	Extent of reaction^c^ (% of IO_3_^–^ reduced to I^–^)^d^
Abiotic	0 ± 0 (0)	0 ± 0 (0)	0 ± 0 (0)	0 ± 0 (0)
MR-1	412.7 ± 77.0 (100)	55.5 ± 0.2 (100)	379.8 ± 7.2 (100)	51.6 ± 3.9 (100)
Δ*mtrA*	28.6 ± 14.1 (7)	4.1 ± 0.7 (7)	286.9 ± 36.8 (76)	35.9 ± 2.8 (70)
Δ*mtrA*-Δ*mtrDEF*	157.2 ± 8.2 (38)	13.2 ± 1.0 (24)	219.3 ± 1.0 (58)	29.0 ± 1.1 (56)
Δ*mtrA*-Δ*dmsEF*	70.2 ± 10.3 (17)	8.3 ± 1.1 (15)	90.2 ± 43.1 (24)	10.2 ± 2.6 (20)
Δ*mtrA*-Δ*SO4360*	143.1 ± 38.0 (35)	15.7 ± 1.1 (28)	238.2 ± 45.5 (63)	31.5 ± 0.7 (61)

*^a^Values represent means of triplicate samples; error represents one standard deviation.*

*^b^Reaction rate was calculated from the first 4-h anaerobic incubation. Cells corresponding to OD = 0.1 contain 50 mg protein.*

*^c^Extent of reaction is reported as the percentage of IO_3_^–^ reduced to I^–^ upon completion of the 24-h incubation period, after which further IO_3_^–^ reduction was minimal.*

*^d^The values in parentheses are in comparison with the wild-type rates (percent) within each set of lactate or formate values.*

The Δ*mtrA*Δ*mtrDEF* and Δ*mtrA*Δ*SO4360* mutant strains provided with formate as the electron donor displayed moderately impaired (between 51 and 70% of wild-type activity) IO_3_^–^ reduction activities when compared to wild-type rates (58 and 63% of the wild-type rate and 56 and 61% of the wild-type extents of reaction, respectively; Table 1 and Figure 2). The Δ*mtrA* mutant displayed a similar profile (between 71 and 102% of wild-type activity) with the wild-type strain (76% of the wild-type rate and 70% of the wild-type extent of reaction, respectively; Table 1 and Figure 2), while the Δ*mtrA*Δ*dmsEF* mutant strain was severely impaired in IO_3_^–^ reduction activity with formate as the electron donor (24% of the wild-type rate and 20% of the wild-type extent of reaction, respectively; Table 1 and Figure 2). These results indicate that EEC component DmsE, but not MtrA, and OMP component DmsF, but not MtrB, are required for IO_3_^–^ reduction with formate as the electron donor (Figure 1).

### Replacement of Mo With W in Defined Minimal Growth Medium and the Effect on IO_3_^–^ Reduction Activity of *Shewanella oneidensis*

**FIGURE 3:**
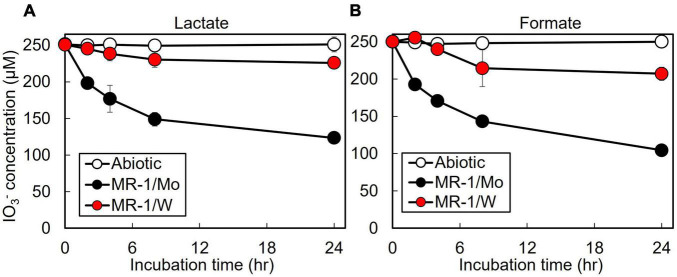
Effect of molybdenum (Mo) substitution with tungsten (W) on IO_3_^–^ reduction activity of *Shewanella oneidensis* with IO_3_^–^ as the electron acceptor and **(A)** lactate or **(B)** formate as the electron donor. Values are means of triplicate samples from anaerobic incubations. Error bars represent one standard deviation.

The *S. oneidensis* DMSO reductase DmsAB is composed of the molybdopterin-binding subunit DmsA and the ferredoxin subunit DmsB, which contains Mo and [4Fe-4S] clusters as co-factors, respectively (Figure 1; Gralnick et al., 2006). To test the hypothesis that *S. oneidensi*s employs DmsAB as the IO_3_^–^ terminal reductase we attempted to generate a Δ*dmsA* deletion mutant, as DmsA is the active component of DMSO reductase, but were unsuccessful. A previous study reported the similar inability to produce a Δ*dmsA* deletion mutant, which indicates that the *dmsA* deletion may be lethal (Gralnick et al., 2006). Mo is the critical catalytic element of the molybdopterin-binding DMSO reductase family, which includes DMSO reductase, nitrate reductase, and formate dehydrogenase (May et al., 1988; Hanzelmann and Mayer, 1998; McEwan et al., 2002; Waite and Trucesdale, 2003). W readily replaces Mo in molybdopterin-binding enzymes, yet equimolar Mo substitution with W results in loss of enzymatic activity of DMSO reductase family enzymes (May et al., 1988; Hanzelmann and Mayer, 1998; Hille, 2002; Waite and Trucesdale, 2003). The substitution of Mo with W did not affect cell fitness (Supplementary Figure 1). Depleting the wild-type strain MR-1 of Mo caused catalytic inactivation of the DmsA subunit and effectively generated a mutant strain deficient in DmsA catalytic activity. After growth in W-containing defined minimal medium, wild-type *S. oneidensis* reduced IO_3_^–^ at severely impaired rates when incubated with either lactate or formate as the electron donor (62 and 42 nmol hr^–1^ mg protein^–1^, respectively), corresponding to only 17 and 11% of the rates measured after growth in Mo-containing defined minimal medium (lactate, 371 nmol hr^–1^ mg protein^–1^; formate, 366 nmol hr^–1^ mg protein^–1^, respectively) (Figure 3 and Table 2). These findings indicate that Mo is required for IO_3_^–^ reduction with either lactate or formate as electron donor, potentially as the critical element of the molybdopterin-binding co-factor of DMSO reductase.

**TABLE 2:** Effect of replacement of molybdenum (Mo) with tungsten (W) on IO_3_^–^ reduction activities of *Shewanella oneidensis*.^a^

Condition or strain	Electron donor: lactate		Electron donor: formate	
		
	IO_3_^–^ reduction rate^b^ (nmol hr^–1^ mg protein^–1^)^d^	Extent of reaction^c^ (% of IO_3_^–^ reduced to I^–^)^d^	IO_3_^–^ reduction rate^b^ (nmol hr^–1^ mg protein^–1^)^d^	Extent of reaction*^c^* (% of IO_3_^–^ reduced to I^–^)^d^
Abiotic	0 ± 0 (0)	0 ± 0 (0)	0 ± 0 (0)	0 ± 0 (0)
MR-1 with Mo	370.9 ± 92.4 (100)	50.8 ± 2.0 (100)	366.0 ± 11.9 (100)	58.2 ± 2.3 (100)
MR-1 with W	62.0 ± 9.4 (17)	9.0 ± 4.2 (18)	42.0 ± 1.0 (11)	17.2 ± 3.6 (30)

*^a^Values represent means of triplicate samples; error represents one standard deviation.*

*^b^Reaction rate was calculated from the first 4-h anaerobic incubation. Cells corresponding to OD = 0.1 contain 50 mg protein.*

*^c^Extent of reaction is reported as the percentage of IO_3_^–^ reduced to I^–^ upon completion of the 24-h incubation period, after which further IO_3_^–^ reduction was minimal.*

*^d^The values in parentheses are in comparison with the presence of Mo (percent) within each set of lactate or formate values.*

### IO_3_^–^ Reduction Activity of Δ*dmsB* Mutant With Lactate or Formate as Electron Donor

**FIGURE 4:**
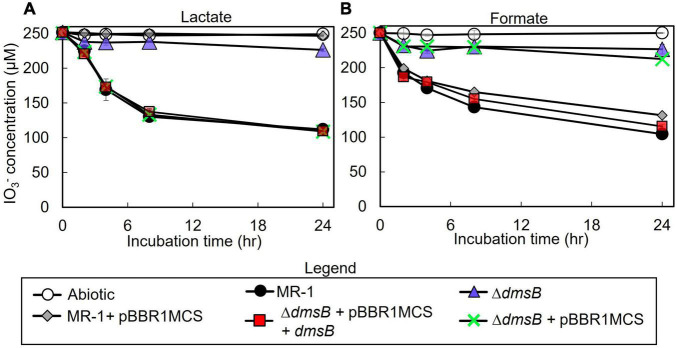
IO_3_^–^ reduction activity of *Shewanella oneidensis* wild-type (MR-1) and Δ*dmsB*, Δ*dmsB* + pBBR*dmsB*,Δ*dmsB* + pBBR1MCS, MR-1 + pBBR1MCS strains with IO_3_^–^ as the electron acceptor and **(A)** lactate or **(B)** formate as the electron donor and their IO_3_^–^ reduction rate. Values are means of triplicate samples from anaerobic incubations. Error bars represent one standard deviation.

**FIGURE 5:**
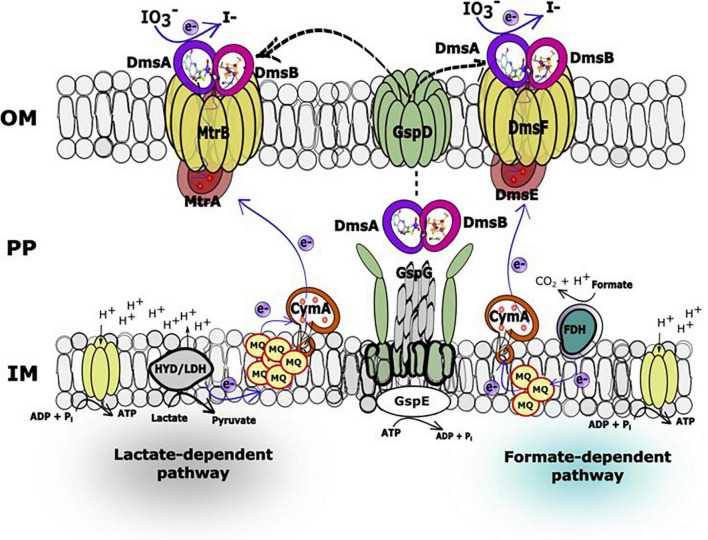
Hypothetical working model of the lactate (MtrAB)- and formate (DmsEF)-dependent IO_3_^–^ reduction electron transport pathways in *Shewanella oneidensis.* In the IO_3_^–^ reduction pathways, electrons originating from lactate dehydrogenase or formate dehydrogenase located at the head end of the electron transport chain are transferred to the inner membrane-localized menaquinone pool and subsequently to CymA, which facilitates electron transfer across the periplasmic space to decaheme *c*-type cytochromes MtrA or DmsE, respectively. At this location in the electron transport chain, the IO_3_^–^ reduction pathways diverge to MtrAB (lactate as electron donor) or DmsEF (formate as electron donor) and terminate with DmsA and DmsB, both of which associate with MtrA (or DmsE) and β-barrel protein MtrB (or DmsF). DmsA and DmsB are both secreted extracellularly by the type II protein secretion system to form a ternary complex with the MtrAB (or DmsEF) EEC modules on the outside face of the outer membrane (Gralnick et al., 2006).

To test the hypothesis that *S. oneidensi*s employs DmsAB as the IO_3_^–^ terminal reductase, we generated a Δ*dmsB* gene deletion mutant. In a previous study, mutant strain Δ*dmsB* was unable to grow anaerobically with DMSO as a terminal election acceptor (Gralnick et al., 2006). Fumarate reduction was not impaired by Δ*dmsB* deletion, which indicates that overall fitness is unaffected (Gralnick et al., 2006). In this study, Δ*dmsB* was also unable to grow with DMSO as electron acceptor and formate as the electron donor, while a Δ*dmsB* transconjugant strain provided with a wild-type copy of *dmsB* (Δ*dmsB* + pBBR1MCS-*dmsB*) grew at wild-type rates with DMSO as electron acceptor (Supplementary Figure 2). Δ*dmsB* was also severely impaired in IO_3_^–^ reduction activity with lactate or formate as the electron donor (18 and 30% of the wild-type rate and 18 and 16% of the wild-type extents of reaction with lactate or formate, respectively; Figure 4 and Table 3), while the Δ*dmsB* + pBBR1MCS-*dmsB* transconjugant strain reduced IO_3_^–^ at wild-type rates and extents of reaction (92 and 85% of the wild-type rate and 102 and 93% of the wild-type extents of reaction with lactate or formate, respectively; Figure 5 and Table 3). As expected, control MR-1 + pBBR1MCS (96 and 80% of the wild-type rate and 102 and 81% of the wild-type extents of reaction with lactate or formate, respectively; Figure 5 and Table 3) reduced IO_3_^–^ at near-wild-type rates, and control Δ*dmsB* + pBBR1MCS (3 and 23% of the wild-type rate and 4 and 26% of the wild-type extents of reaction with lactate or formate, respectively; Figure 5 and Table 3) was severely affected. These results further indicate that DmsAB displays broad substrate specificity and reduces IO_3_^–^ as an alternate terminal electron acceptor.

**TABLE 3:** IO_3_^–^ reduction activities of wild-type and *dmsB* mutant strains of *Shewanella oneidensis*.^a^

Condition or strain	Electron donor: lactate		Electron donor: formate	
		
	IO_3_^–^ reduction rate^b^ (nmol hr^–1^ mg protein^–1^)^d^	Extent of reaction^c^ (% of IO_3_^–^ reduced to I^–^)^d^	IO_3_^–^ reduction rate^b^ (nmol hr^–1^ mg protein^–1^)^d^	Extent of reaction^c^ (% of IO_3_^–^ reduced to I^–^)^d^
Abiotic	0 ± 0 (0)	0 ± 0 (0)	0 ± 0 (0)	0 ± 0 (0)
MR-1	412.7 ± 77.0 (100)	55.5 ± 0.2 (100)	366.0 ± 11.9 (100)	58.2 ± 5.7 (100)
Δ*dmsB*	72.9 ± 18.9 (18)	10.0 ± 1.0 (18)	108.2 ± 13.2 (29.5)	9.4 ± 1.4 (16.2)
MR-1 + pBBR1MCS	395.3 ± 36.0 (96)	56.4 ± 2.7 (102)	294.3 ± 21.7 (80)	47.5 ± 4.4 (81.2)
Δ*dmsB* + pBBR1MCS	11.3 ± 20.5 (3)	2.0 ± 1.1 (4)	84.0 ± 13.3 (22.7)	15.0 ± 8.2 (25.8)
Δ*dmsB* + pBBR1MCS-*dmsB*	381.5 ± 9.2 (92)	56.5 ± 0.2 (102)	311.3 ± 16.7 (85)	53.9 ± 3.2 (92.6)

*^a^Values represent means of triplicate samples; error represents one standard deviation.*

*^b^Reaction rate was calculated from the first 4-h anaerobic incubation. Cells corresponding to OD = 0.1 contain 50 mg protein.*

*^c^Extent of reaction is reported as the percentage of IO_3_^–^ reduced to I^–^ upon completion of the 24-h incubation period, after which further IO_3_^–^ reduction was minimal.*

*^d^The values in parentheses are in comparison with the wild-type rates (percent) within each set of lactate or formate values.*

## Discussion

The *S. oneidensis* genome encodes four EECs, each composed of three major components: periplasmic electron carrier (PEC), outer membrane β-barrel protein (OMP), and extracellular terminal reductase (ETR) (Figure 1). *S. oneidensis* EECs are involved in the reduction of Fe(III), Mn(IV), Mn(III), flavins, and DMSO (Bucking et al., 2010; Coursolle and Gralnick, 2010; Szeinbaum et al., 2014). Several EEC components are interchangeable and can functionally replace the corresponding paralog proteins (Coursolle and Gralnick, 2010, 2012; Schicklberger et al., 2013). *S. oneidensis* PECs include the decaheme *c*-type cytochromes MtrA, MtrD, DmsE, and SO4360 (Bucking et al., 2010; Coursolle and Gralnick, 2010; Schicklberger et al., 2013). MtrA is the primary PEC of the *S. oneidensis* Fe(III), Mn(IV), Mn(III), and flavin reduction systems. The overexpression of MtrD restores wild-type Fe(III)-citrate reduction rates to an Δ*mtrA* mutant (Bucking et al., 2010; Coursolle and Gralnick, 2010; Schicklberger et al., 2013). DmsE is primarily devoted to DMSO reduction (Gralnick et al., 2006; Bucking et al., 2010), but partially restores Fe(III)-citrate reduction to mtrA Mutants (Bucking et al., 2010; Coursolle and Gralnick, 2010; Schicklberger et al., 2013). SO4360 displays high amino acid sequence homology to other *S. oneidensis* PECs, but has not yet been assigned a respiratory function (Bucking et al., 2010; Coursolle and Gralnick, 2010; Schicklberger et al., 2013).

The four *S. oneidensis* ETRs are involved in Fe(III) or DMSO reduction and include the decaheme *c*-type cytochromes MtrC, MtrF, and OmcA. MtrF and OmcA functionally replace MtrC (Coursolle and Gralnick, 2010), while MtrDE only partially replaces MtrAB in the Fe(III)-citrate reduction pathways (Coursolle and Gralnick, 2012). Although DmsEF are the paralogs of MtrAB, DmsEF does not functionally replace MtrAB in the Fe(III)-citrate reduction pathway (Coursolle and Gralnick, 2012). The third and fourth ETRs (DmsAB and SO4358/SO4357) each contain both molybdopterin and 4Fe-4S clusters as co-factors, respectively (Gralnick et al., 2006; Bucking et al., 2010; Coursolle and Gralnick, 2010; Schicklberger et al., 2013). OMPs form a pore-like structure through the outer membrane that directs electron transfer between the PEC and ETR components (Beliaev and Saffarini, 1998; Hartshorne et al., 2009). The *S. oneidensis* genome harbors four OMP paralogs (MtrB, MtrE, DmsF, and SO4359) (Bucking et al., 2010; Coursolle and Gralnick, 2010; Schicklberger et al., 2013). SO4359, a paralog of DmsE, functionally replaces MtrB under Fe(III) citrate-reducing conditions (Schicklberger et al., 2013). The MtrAB module of MtrCAB is required for IO_3_^–^ reduction by *S. oneidensis* with lactate (but not formate) as the electron donor (Toporek et al., 2019). Similar electron donor-dependent respiratory phenotypes of *S. oneidensis* were also previously reported with technetium [Tc(VII)] as the terminal electron acceptor (Payne and DiChristina, 2006). Based on these previous findings, we hypothesized that *S. oneidensi*s employed an ETR other than MtrC to deliver electrons to IO_3_^–^.

In our previous report, a lactate (MtrAB)-dependent *S. oneidensis* IO_3_^–^ reduction system was proposed (Toporek et al., 2019). In this working model, electrons originating from lactate dehydrogenase were transported *via* the menaquinone pool, CymA, and MtrAB to the unknown terminal IO_3_^–^ reductase that was translocated to the outside face of the outer membrane *via* type II protein secretion, while IO_3_^–^ reduction with formate as the electron donor was MtrABC-independent (Toporek et al., 2019). In the expanded working model with formate as the electron donor, electrons originating from formate dehydrogenase located at the head end of the electron transport chain are transferred to the inner membrane-localized menaquinone pool and subsequently to CymA (Toporek et al., 2019), which facilitates electron transfer across the periplasmic space to DmsE, a decaheme *c*-type cytochrome partially embedded in the outer membrane and encased in the β-barrel protein DmsF (Figure 5). DmsA and DmsB are secreted extracellularly by the type II protein secretion system and form a ternary complex with the DmsEF PEC module on the outside face of the outer membrane (Gralnick et al., 2006). The extracellular DMSO reductase DmsAB of *S. oneidensis* reduces IO_3_^–^ as an alternate terminal electron acceptor. The SO4358/4357 complex, a paralog of DmsAB (Figure 1), is unlikely to be the preferred or alternate IO_3_^–^ terminal reductase with lactate or formate as the electron donors.

The lactate-dependent *S. oneidensis* IO_3_^–^ reduction system utilizes MtrAB and does not require DmsEF, but does require DmsAB as the IO_3_^–^ terminal reductase. DmsAB is evolutionarily unrelated to MtrC, MtrF, or OmcA. Although the Mtr respiratory pathway is modular, MtrAB has not been reported to transfer electrons to extracellular reductases apart from MtrC, MtrF, or OmcA. However, previous work which tested all possible combinations of Mtr paralogs (displayed in Figure 1) were tested solely with Fe(III)-citrate as an electron acceptor (Schicklberger et al., 2013), and have not been tested with most other electron acceptors including IO_3_^–^. Compellingly, DmsE functionally replaces MtrA (Coursolle and Gralnick, 2012), and the DmsF paralog SO4359 functionally replaces MtrB under Fe(III) citrate-reducing conditions (Schicklberger et al., 2013). Furthermore, MtrAB forms a stable complex in the outer membrane without MtrC, and redox properties of MtrA are modulated upon the formation of an MtrCAB complex (Hartshorne et al., 2009). DmsAB may hypothetically localize to the outer face of the outer membrane through the type II secretion porin GspD and pair with MtrAB to reduce extracellular IO_3_^–^ with lactate as the electron donor.

The extracellular IO_3_^–^ reductase system of *S. oneidensis* differs from the periplasmic IO_3_^–^ reductase (Idr) system of *Pseudomonas* sp. strain SCT and *Denitromonas* sp. IR-12 homologs that display amino acid sequence homology to respiratory arsenite oxidase; however, both systems require molybdopterin coordinating Mo (Yamazaki et al., 2020; Reyes-Umana et al., 2022). Results of this study provide new insights into the molecular mechanism of microbial IO_3_^–^ reduction, yield details important to the biogeochemical cycling of iodine in marine systems, and provide information crucial to the development of alternative bioremediation technologies for the treatment of radioactive iodine-contaminated subsurface environments.

## Data Availability Statement

The original contributions presented in the study are included in the article/Supplementary Material, further inquiries can be directed to the corresponding author.

## Author Contributions

H-DS, YT, JM, and RM performed the experiments. H-DS, YT, and TD wrote the manuscript. All authors had given approval to the final version of the manuscript.

## Author Disclaimer

Any opinion, findings, and conclusions or recommendations expressed in this material are those of the authors and do not necessarily reflect the views of the US Department of Energy Office of Environmental Management.

## Conflict of Interest

The authors declare that the research was conducted in the absence of any commercial or financial relationships that could be construed as a potential conflict of interest.

## Publisher’s Note

All claims expressed in this article are solely those of the authors and do not necessarily represent those of their affiliated organizations, or those of the publisher, the editors and the reviewers. Any product that may be evaluated in this article, or claim that may be made by its manufacturer, is not guaranteed or endorsed by the publisher.
