# Supplementary material for: Iodate Reduction by Shewanella oneidensis Requires Genes Encoding an Extracellular Dimethylsulfoxide Reductase
Source: Front Microbiol. 2022 Apr 14;13:852942. doi: 10.3389/fmicb.2022.852942 (PMC9048795; doi:10.3389/fmicb.2022.852942)
Supplement: Supplementary file 1 [file Data_Sheet_1.DOCX]

M1 minimal medium composition

| KH_2_PO_4_ | 22.05 | mM |
| --- | --- | --- |
| K_2_HPO_4_ | 37.95 | mM |
|  |  |  |
| CoSO_4_×7H_2_O | 0.50 | μM |
| NiCl_2_×6H_2_O | 0.83 | μM |
| NaCl | 0.99 | μM |
|  |  |  |
| H_3_BO_3_ | 452.85 | nM |
| ZnSO_4_×7H_2_O | 8.35 | nM |
| Na_2_MoO_4_×2H_2_O *or* Na_2_WO_4_×2H_2_O | 31.00 | nM |
| CuSO_4_×5H_2_O | 1.68 | nM |
| MnSO_4_×H_2_O | 10.06 | nM |
|  |  |  |
| MgSO_4_×7H_2_O | 811.42 | μM |
| CaCl_2_×2H_2_O | 387.73 | μM |
| EDTA, disodium salt | 59.13 | μM |
| FeSO_4_×7H_2_O | 4.32 | μM |
|  |  |  |
| L-serine | 19.03 | μM |
| L-arginine | 11.48 | μM |
| L-glutamic acid | 13.59 | μM |
|  |  |  |
| Na-lactate *or* Na-formate | 20.00 | mM |
| NaSeO_4_ | 11.50 | μM |
| (NH_4_)_2_SO_4_ | 12.65 | mM |
| HEPES | 10.00 | mM |
| FeCl_3_ | 100.00 | μM |
|  |  |  |
| NaIO_3_^-^ | 250.00 | μM |

**Table S1.** Bacterial strains and plasmids used in this study.

| **Strain** | **Features** | **Source** | |
| --- | --- | --- | --- |
| ***S. oneidensis*** |  | |  |
| MR-1 | Wild-type strain | | ATCC |
| ∆*mtrA* | In-frame m*trA* deletion mutant | | (Szeinbaum *et al.*, 2014)^47^ |
| ∆*mtrA-*∆*mtrDEF* | In-frame *mtrA* and *mtrDEF* deletion mutant | | This study |
| ∆*mtrA-*∆*dmsEF* | In-frame *mtrA* and *dmsEF* deletion mutant | | This study |
| ∆*mtrA-*∆*SO4360* | In-frame *mtrA* and *SO4360* deletion mutant | | This study |
| ∆*dmsB* | In-frame *dmsB* deletion mutant | | This study |
| ∆*dmsB-dmsB^+^* | ∆*dmsB* complemented with pBBR-*dmsB* | | This study |
| ***E. coli*** |  | |  |
| EC100D *pir*-116 | *F- mcrA Δ(mrr-hsdRMS-mcrBC) φ80dlacZΔM15 ΔlacX74 recA1 endA1 araD139 Δ(ara, leu)7697 galU galK λ- rpsL (StrR) nupG pir-116(DHFR)* | | Epicentre |
| β2155 λ *pir* | *thrB1004 pro thi strA hsdS lacZ_M15(F9 lacZ∆M15 laclq traD36 proA1 proB1) ∆dapA::erm pir::RP4 Km^R^* | | (Dehio *et al.*, 1997)^60^ |
| **Plasmids** |  | |  |
| pKO2.0 | In-frame gene deletion vector  4.5 kb γR6K, *mob*RP4 *sacB* Gm^R^ *lacZ* | | (Burns *et al.*, 2009)^59^ |
| pBBR1MCS | Broad-host range vector, Cm^R^, lacZ | | (Kovach *et al.,*1995)^58^ |
| pBBR-*dmsB* | pBBR1MCS containing wild-type  copy of *dmsB* | | This study |

**Table S2.** Primers used in this study

| **Primer** | | **Sequence (5' to 3')** | **Remark** | |
| --- | --- | --- | --- | --- |
| ***∆mtrDEF*** | | | |  |
| D1 | ACTAGTGGATCCTGCCGTCGACACGTTGT | | | BamHI(underlined) |
| D2 | GGGCCTGCATCATCGAGTTTTCCCGAGTGCGTTTTATTT | | |  |
| D3 | AAATAAAACGCACTCGGGAAAACTCGATGATGCAGGCCC | | |  |
| D4 | GGTACCCTCGAGTGAATGCCTTCTGTTTTACCTGT | | | XhoI(underlined) |
| TF | ACAGCTCACGTAGCTGGGC | | |  |
| TR | ATGCACAGTTTTCAATATCGATACG | | |  |
| ***∆dmsEF*** | | | |  |
| D1 | ACTAGTGGATCCCACTCCTTATATCTATCACTTTTCGAA | | | BamHI(underlined) |
| D2 | GTTCCATTGTCTTTACCTCTTATCAAATTTAATGCCTTATGGTTAACATGAC | | |  |
| D3 | GTCATGTTAACCATAAGGCATTAAATTTGATAAGAGGTAAAGACAATGGAAC | | |  |
| D4 | GAGTCCGTCGACTTGGAATGAGTATCGACCCAT | | | SalI(underlined) |
| TF | TATCATGGATACCTTACTGCGC | | |  |
| TR | ATATTGAGTCTTTTTAGGATCGCC | | |  |
| ***∆SO4360*** | | | |  |
| D1 | ACTAGTGGATCCAGGCTGCTAAATGATAAAAGTACATTAGA | | | BamHI(underlined) |
| D2 | CGTTTTACTTAACTTCATTACTCACTCCACCATGGGCTGAATTAAAAAAACT | | |  |
| D3 | AGTTTTTTTAATTCAGCCCATGGTGGAGTGAGTAATGAAGTTAAGTAAAACG | | |  |
| D4 | GAGTCCGTCGACCATCTTGTTGCTGAACACCTCTT | | | SalI(underlined) |
| TF | TGCAGAATTGACTAAGTTACAATTAGA | | |  |
| TR | TGAGTATTGAACCTATGATCAATTG | | |  |
| ***∆dmsB*** | | | |  |
| D1 | TAGCTC ACTAGT AGGTAAAGATGAAGCGGC | | | SpeI(underlined) |
| D2 | AGCCCCCCACATTTCACTTTTCATCTACCCCTTATGCC | | |  |
| D3 | GGCATAAGGGGTAGATGAAAAGTGAAATGTGGGGGGCT | | |  |
| D4 | GAGTCCGTCGACTGCTGTCACAAACGTCATG | | | SalI(underlined) |
| TF | TAGTCAATGATAGCTATGCA | | |  |
| TR | AGCAGCCAAACTCAATTG | | |  |
| **pBBR1MCS-*dmsB*** | | | |  |
| dB-F | CTGATAGGTACCATGACTCAACAAACACAATATGGTTT | | | KpnI(underlined) |
| dB-R | TCGATCTCTAGATTACACTTCTGCAGGGTTTAATAATTG | | | XbaI(underlined) |

**Figure S1.**


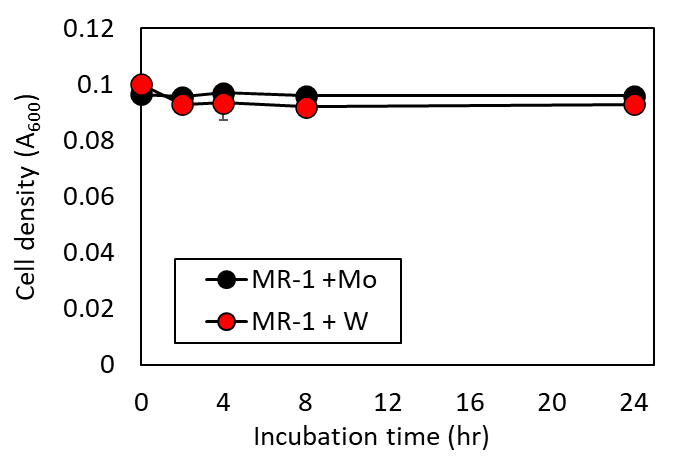


Effect of molybdenum (Mo) substitution with tungsten (W) on cell density over time of *S. oneidensis* with IO_3_^−^ as the electron acceptor and formate as the electron donor. Values are means of triplicate samples from anaerobic incubations. Error bars represent one standard deviation.

**Figure S2.**


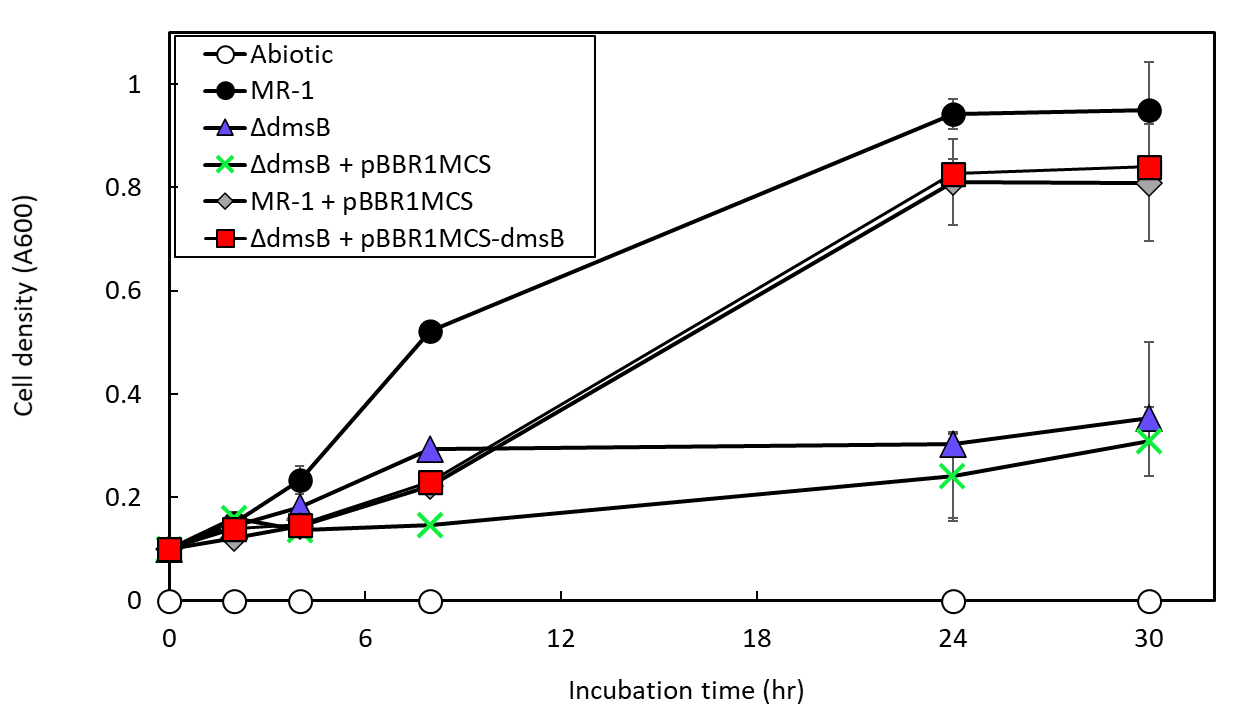


Cell density over time of *S. oneidensis* wild-type (MR-1) and *ΔdmsB*, *ΔdmsB+*pBBR*dmsB, ΔdmsB+*pBBR1MCS*,* MR-1+pBBR1MCS strains with formate as the electron donor and DMSO as electron acceptor. Values are means of triplicate samples from anaerobic incubations. Error bars represent one standard deviation.

**Figure S3.**


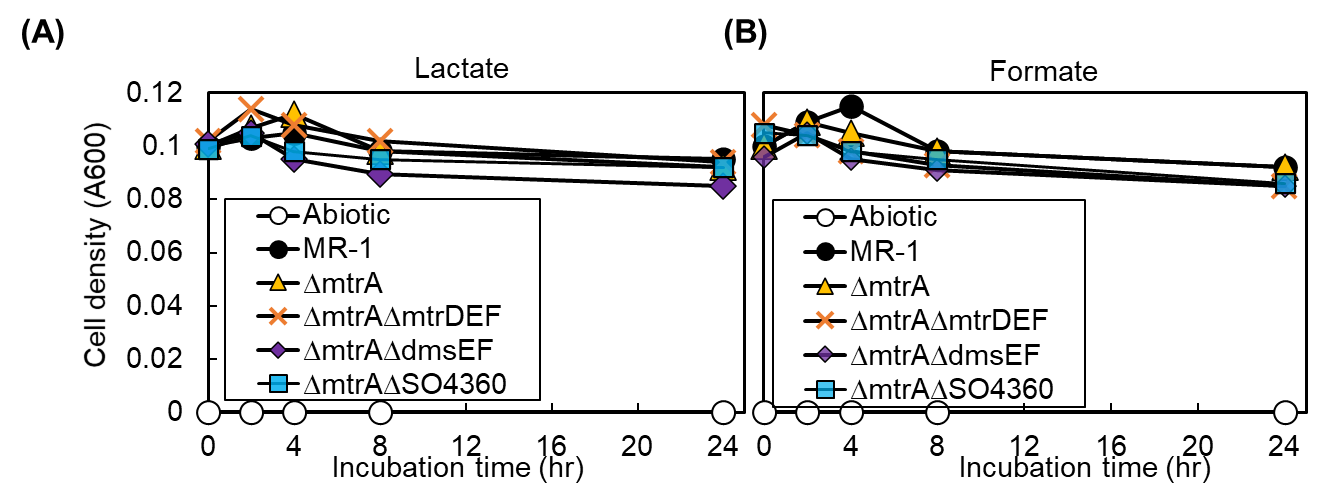


Cell density over time of *S. oneidensis* wild-type (MR-1) and *ΔmtrA*, *ΔmtrAΔmtrDEF, ΔmtrAΔdmsEF,* and *ΔmtrAΔSO4360* mutants with IO_3_^−^ as the electron acceptor and **(A)** lactate or **(B)** formate as electron donor. Values are means of triplicate samples from anaerobic incubations. Error bars represent one standard deviation.
